# Editorial: Bacterial Exotoxins: How Bacteria Fight the Immune System

**DOI:** 10.3389/fimmu.2016.00300

**Published:** 2016-08-02

**Authors:** Inka Sastalla, Denise M. Monack, Katharina F. Kubatzky

**Affiliations:** ^1^Bacterial Pathogenesis Unit, Laboratory of Clinical Infectious Diseases, National Institute of Allergy and Infectious Diseases, National Institutes of Health, Bethesda, MD, USA; ^2^Department of Microbiology and Immunology, Stanford School of Medicine, Stanford University, Stanford, CA, USA; ^3^Zentrum für Infektiologie, Medizinische Mikrobiologie und Hygiene, Universitätsklinikum Heidelberg, Heidelberg, Germany

**Keywords:** bacterial toxins, immune evasion, infectious diseases, innate immune response, adaptive immunity, super antigen, bacterial effector protein, quorum sensing

**The Editorial on the Research Topic**

**Bacterial Exotoxins: How Bacteria Fight the Immune System**

Upon infection with a bacterial pathogen, the body initiates both innate and adaptive immune responses with the ultimate goal to eliminate the invader and to return to homeostasis. Occasionally, however, the body may react inadequately, resulting in collateral damage to tissues if the response it too strong or in a failure to eradicate the pathogen if the response is too weak or ephemeral. Functionally diverse toxins released by bacteria during infection can contribute considerably to the outcomes of the immune response. For example, bacterial toxins may mediate bacterial evasion of immune recognition, facilitate dwelling within protected niches of eukaryotic cells, or modulate pro-inflammatory responses. Furthermore, in recent years, it has become evident that beyond their canonical actions, bacterial toxins may initiate other cellular responses. For example, besides inducing cytolysis, pore-forming toxins may also induce autophagy, pyroptosis, or activation of the MAPK pathways, resulting in adjustment of the host immune response to infection and modification of inflammatory responses both locally and systemically (1, 2).

Exotoxins can be single polypeptides or heteromeric protein complexes that act on different parts of the cells. At the cell surface, they may insert into the membrane to cause damage, bind to receptors to initiate their uptake, or facilitate interactions with other cell types. For intracellular activity, exotoxins need to be translocated across the eukaryotic membrane. Gram-negative bacteria can directly inject effector proteins in a receptor-independent manner by use of specialized needle apparatus such as bacterial type II, type III, or type IV secretion systems. Other methods of translocation include the phagocytic uptake of bacteria followed by toxin secretion and receptor-mediated endocytosis. Receptor-based uptake allows the targeting of distinct cell types uniquely expressing the receptor. It is initiated by the binding of heteromeric toxin complexes to cell surfaces and is completed by the translocation of the effector proteins across the endosomal membrane. Once in the cytosol, toxins interact with specific eukaryotic target proteins to cause post-translational modifications of host proteins that often result in the manipulation of cellular signaling cascades and inflammatory responses (3).

The intention of this special issue on bacterial exotoxins is to gather current knowledge on the interaction of these versatile effector proteins with the host immune system and to describe mechanisms of immune modification and evasion. We thank the authors of the following 16 articles for providing diverse overviews, comprehensive reviews, and intriguing new data regarding the effects on, and interactions with, three important groups of immune function: (1) barrier cells such as fibroblasts, and epithelial and endothelial cells that are responsible for mediating local immune responses, (2) innate phagocytic cells, and (3) cells of the adaptive arm of immunity.

The first group of manuscripts addresses the interaction of bacterial toxins of both Gram-positive and Gram-negative bacteria with initial barrier cells. Mayer et al. explore the effects of Shiga Toxin (Stx) expressed by enterohemorrhagic *E. coli* on renal endothelial and epithelial cells. In the kidneys, Stx causes hemolytic uremic syndrome, which can result in renal failure. The authors found that Stx-induced damage of renal cells stimulates the release of host-derived damage-associated molecular patterns (DAMPs), such as histones or high-mobility group protein B1 (HMGB1), and that these contribute to the severity of the disease. Ashida et al. review how *Shigella*, a pathogen of the intestinal tract that causes dysentery, uses effector proteins that are injected into host target cells *via* a type-III secretion system to induce cell death, to modulate protein trafficking and signaling, and to ultimately interfere with both innate and adaptive immunity. Moreover, the poultry pathogen *Salmonella pullorum* expresses an iron-storage protein called bacterioferritin that appears to be a major antigen for the chicken humoral response to *S. pullorum* infection (Xu et al.). Using a chicken fibroblast cell line, the authors furthermore identify the induction of the type I interferon IFN-β as a major consequence of bacterioferritin exposure. The last article on the effects of toxin on host barrier function is by von Hoven et al. who studied the pore-forming α-toxin of *Staphylococcus aureus*. Using mouse embryonic fibroblasts, the authors show that stress-induced basal phosphorylation of the eukaryotic translation initiation factor 2α (eIF2α) results in tolerance to the toxin. Macrophages, however, are tolerant to α-toxin independent of such a stress response, underlining the fact that various host cell types can mediate different layers of tolerance and protection.

Phagocytes, such as macrophages, neutrophils, and dendritic cells, provide an immediate response and protection during the early stages of bacterial invasion. Therefore, many bacterial virulence factors and toxins specifically target these innate immune cells. Five articles in this special issue review multiple aspects of macrophage–toxin interactions. While do Vale et al. give a comprehensive overview on how bacteria use toxins as weapons to avoid recognition by phagocytes, Barth et al. focus on the effects of the toxins C3bot of *Clostridium botulinum* and C3lim of *Clostridium limosum*, which exclusively target monocytes and macrophages to ADP-ribosylate cellular Rho GTPases. Because of their cell specificity, the authors propose that engineered inactive variants of these two toxins may potentially be useful as both therapeutic and molecular tools. Greaney et al. review the various interactions between bacterial exotoxins and the inflammasomes, which are intracellular sensors of danger- and pathogen-associated molecular patterns and key to the elicitation of immune responses to a number of pathogens. An additional review by Simon and Hilbi describes how the approximately 300 effector proteins of *Legionella pneumophila*, the causative agent of legionellosis, help to establish a bacterial replication niche in alveolar macrophages. Finally, Zhang et al. investigate the effect of a previously uncharacterized protein of *Streptococcus suis* that signals *via* activation of TLR2, resulting in the production pro-inflammatory cytokines, such as IL-1, MCP-1, and TNF-α.

Neutrophils are highly motile phagocytes that, in addition to the phagocytic uptake and killing of bacteria, can form neutrophil extracellular traps (NETs) to destroy bacteria in a phagocytosis-independent manner. In a comprehensive mini-review, von Köckritz-Blickwede et al. give an overview on the modulation of NET formation by microbial virulence factors. Similarly, Uchiyama et al. describe how the pore-forming toxin streptolysin O of *Streptococcus pyogenes* manipulates neutrophil activity and renders the bacterium resistant to neutrophil killing by impairing NET formation, extracellular killing, and the oxidative burst reaction of neutrophils. Maurer et al. describe the interesting capacity of a bitter receptor expressed on neutrophils to recognize bacterial quorum sensing (QS) molecules that bacteria employ for bacterial communication. However, the effect of QS molecules on host cell signaling is not restricted to neutrophils; Liu et al. review how secreted QS molecules of *Pseudomonas aeruginosa* modulate and functionally impair host cells.

Finally, various authors address the interactions of toxins with cells of the adaptive immune system. Bacterial superantigens simultaneously bind to the T cell receptor and to the MHC class II molecule on the surface of antigen-presenting cells (APCs) in a non-specific, antigen-independent manner. The crosslinking causes a massive activation of T cells, ultimately resulting in anergy and the activation of regulatory T cells (Tregs). Krakauer et al. review our current knowledge of danger signaling pathways in T cells that are induced by the superantigen staphylococcal enterotoxin B (SEB) of *S. aureus*, and they propose appealing strategies of how these pathways may be exploited to discover novel drug targets. Sähr et al. investigated whether superantigen-triggered signaling cascades in APCs could provide a microenvironment that facilitates the differentiation of Tregs. However, some toxins can also manipulate T cell differentiation directly and in the absence of an APC. Hildebrand et al. show that the *Pasteurella multocida* Toxin (PMT) can modulate T cell signaling through the activation of specific transcription factors, ultimately resulting in the differentiation of naive T cells into Th17.

In summary, the articles in this special issue highlight the complexity of bacterial toxin interaction with different aspects and cells of the immune system. In particular, it becomes apparent that bacteria have developed intricate means to modify immune cell functions in a precise, strain-specific, and targeted manner, emphasizing the fact that during bacterial infections completely different strategies are used by the invading pathogen to escape the immune system. In light of the increasing occurrences of antibiotic resistances in bacteria observed worldwide, research in recent years has therefore focused on more targeted solutions to fight and prevent infections, such as enhancing host immune functions. A more detailed understanding of how bacteria manipulate the immune system may help to better understand the disease and to ultimately find better therapeutic treatments.

## Author Contributions

All authors listed have made substantial, direct, and intellectual contribution to the work and approved it for publication.

## Conflict of Interest Statement

The authors declare that the research was conducted in the absence of any commercial or financial relationships that could be construed as a potential conflict of interest.
